# Exploring the diverse biomedical applications of programmable and multifunctional DNA nanomaterials

**DOI:** 10.1186/s12951-023-02071-2

**Published:** 2023-08-23

**Authors:** Liuru Fang, Chen Shi, Yuhua Wang, Zuzhao Xiong, Yumei Wang

**Affiliations:** 1 Hubei Province Key Laboratory of Systems Science in Metallurgical Process, Wuhan University of Science and Technology, Wuhan, 430081 China; 2 Department of Pharmacy, Union Hospital, Tongji Medical College, Huazhong University of Science and Technology, Wuhan, 430022 China; 3 Hubei Province Clinical Research Center for Precision Medicine for Critical Illness, Wuhan, 430022 China; 4 Department of Nephrology, Union Hospital, Tongji Medical College, Huazhong University of Science and Technology, Wuhan, 430022 China

**Keywords:** DNA nanomaterials, Biological applications, DNA self-assembly, Tumors

## Abstract

DNA nanoparticles hold great promise for a range of biological applications, including the development of cutting-edge treatments and diagnostic tests. Their subnanometer-level addressability enables precise, specific modifications with a variety of chemical and biological entities, making them ideal as diagnostic instruments and carriers for targeted delivery. This paper focuses on the potential of DNA nanomaterials, which offer scalability, programmability, and functionality. For example, they can be engineered to provide highly specific biosensing and bioimaging capabilities and show promise as a platform for disease diagnosis and treatment. Successful operation of various biomedical nanomaterials has been demonstrated both in vitro and in vivo. However, there are still significant challenges to overcome, including the need to improve the scalability and reliability of the technology, and to ensure safety in clinical applications. We discuss these challenges and opportunities in detail and highlight the progress and prospects of DNA nanotechnology for biomedical applications.

## Introduction

**Fig. 1:**
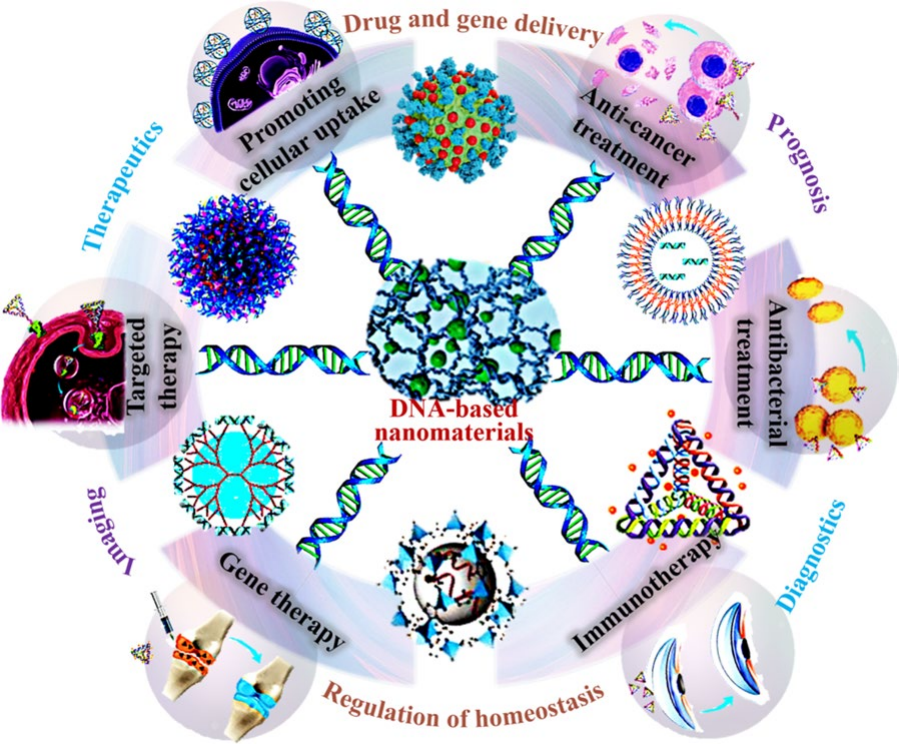
Application of DNA nanomaterials in various aspects of biomedicine

DNA serves as a fundamental building block of all life on Earth, encoding genetic information [1, 2]. However, due to its unique chemical and structural properties, DNA can also be used as a programmable material to construct precise and highly specific artificial nanomaterials [3–6]. The rapid development of DNA nanotechnology has led to wide-ranging applications of DNA nanomaterials in the field of biomedicine, including sensing, diagnosis, treatment, and imaging (Fig. 1). They can be employed as highly sensitive sensors for detecting biomarkers. In the realm of diagnostics, DNA nanomaterials provide a platform for highly specific biomarker detection. As drug carriers, they enable precise drug delivery and release. Additionally, DNA nanomaterials are utilized in gene therapy and high-contrast imaging. These applications offer new approaches and tools for early disease diagnosis and treatment. With ongoing technological advancements, the prospects for DNA nanomaterials in biomedicine remain promising [7–10]. Compared to traditional nanomaterials, DNA nanomaterials possess several distinct advantages. Firstly, they are primarily biocompatible, biodegradable, and non-cytotoxic, as DNA molecules are naturally occurring in living organisms and can be easily recognized by the body. Secondly, their properties can be precisely tailored, and DNA nanomaterials of specific size or shape, such as DNA origami (DO), can be designed and assembled with high accuracy. Thirdly, their surfaces can be programmable modified with molecular-level precision and control, allowing for the specific attachment of various entities, including proteins, nanoparticles, and drugs. Finally, their high sequence specificity makes them ideal for preparing specific biosensors and drug delivery systems, which can enhance therapeutic efficacy while reducing adverse reactions. In addition, researchers have developed 2D and 3D DNA nanomaterials, including DNA nanoporous scaffolds and 3D DNA nanomachines, which have opened up new opportunities and challenges for biomedical research and therapy [11–14]. This review summarizes the latest research on typical DNA nanomaterials, their unique properties, and their applications in biomedicine over the past five years, with a particular emphasis on biosensing, disease diagnosis and treatment, and biological imaging. Furthermore, we discuss the challenges facing DNA nanomaterials in the biomedical field and provide insights into their future development direction.

## DNA nanomaterials

DNA is a versatile polymer material with a flexible conformation that can be precisely designed. It is widely used to construct complex microstructures and nanostructures. DNA nanotechnology has greatly promoted the development of DNA nanomaterials, making them more complex, convenient, and diverse. The advancement of DNA manipulation technology has facilitated the fabrication of DNA-based entities utilizing stiffness as a fundamental parameter. These include DNA tile components and DNA nano-dynamic mechanical components [15, 16], which are used as raw materials for constructing nanostructures of various sizes [17, 18]. The specific base arrangement and superposition of base arrangements can affect the flexibility of DNA. The preparation of DNA nanomaterials is based on the self-assembly capability of DNA molecules and the principle of specific base pairing. The preparation process involves designing and synthesizing DNA sequences, mixing DNA sequences, self-assembly under controlled conditions, regulation and modification of nanostructures, as well as characterization and validation of the nanomaterials, among other key steps. DNA nanomaterials differ significantly from other common materials, exhibiting unique mechanical properties. Firstly, they demonstrate remarkable flexibility, being able to undergo bending and deformation under external stress without breaking or fracturing. This flexibility enables DNA nanoparticles to adapt to various complex environments and application requirements. Secondly, DNA nanoparticles also exhibit good elasticity, meaning they can recover their original shape and maintain the integrity and stability of their structure after being subjected to force. This elastic characteristic allows DNA nanoparticles to resist external pressure and deformation and maintain their functionality and morphology under the restorative force. Additionally, under appropriate conditions, DNA nanoparticles demonstrate high stability, being able to preserve their structure and performance without damage over an extended period. This high stability provides reliability and durability to DNA nanoparticles in various biomedical applications.

Functional nanomaterials are constructed by using various DNA shapes, including round, circular, tetrahedral and branched shapes. Among them, branched DNA offers adjustable size, polyvalence, and controllable symmetry [19–22]. Liu et al. [23] demonstrated the assembly process of various substances based on DNA origami technology. DNA origami serves as a foundational technique in nanomanufacturing for the fabrication of shape-controllable nanomaterials. This technology offers great potential for the development of highly controllable nanomaterials, with extensive applications in fields such as nanoelectronics, photonics, and biomedical engineering.

**Fig. 2:**
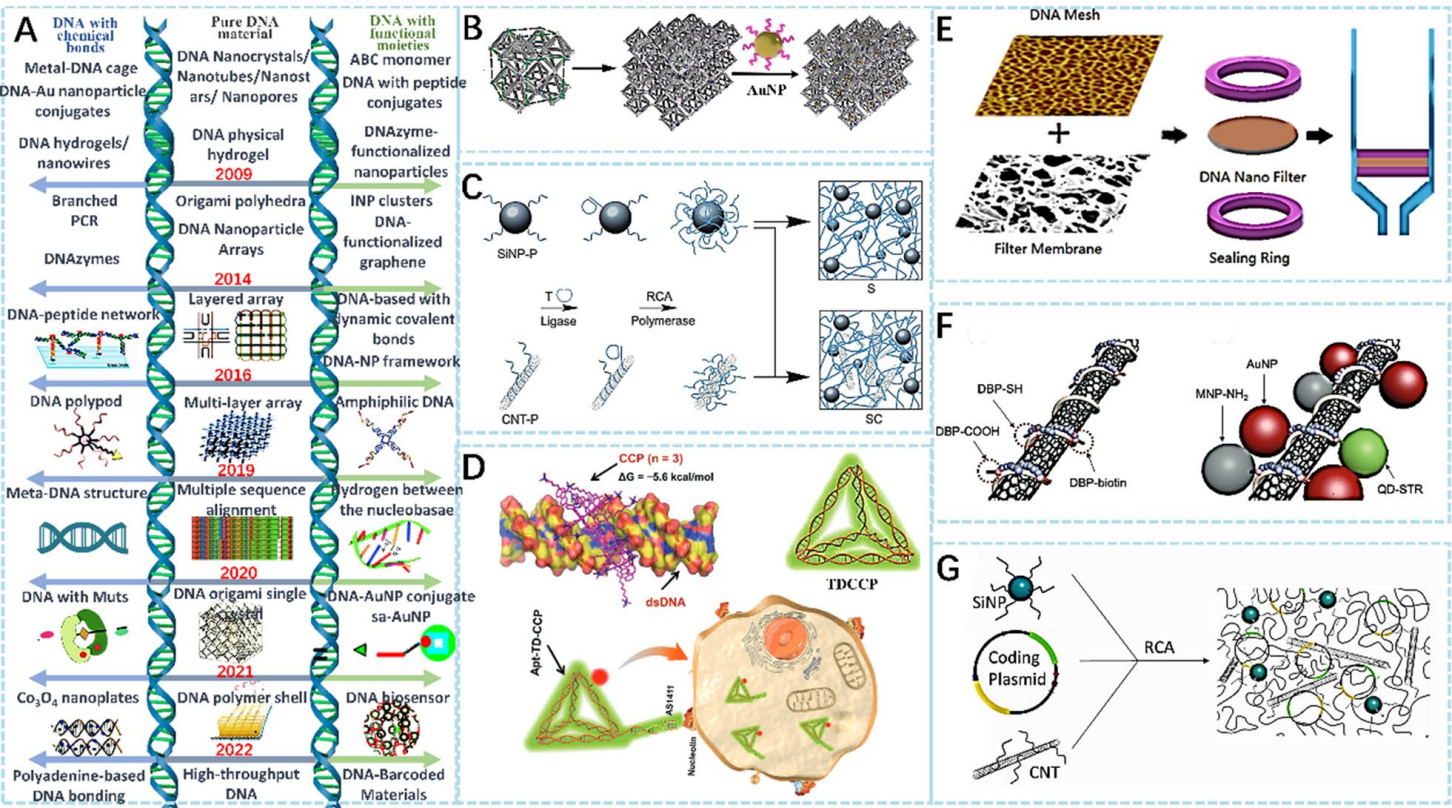
**A** History of branched DNA [25–37]. **B** Assembly of octahedra frames into DNA lattice [11]. **C** Synthesis of binary and ternary SiNP/CNT–DNA nanocomposite materials [38]. **D** Molecular docking study of the potential drug carriers. Stable binding poses of cationic conjugated polymers (CCP) and selective cellular uptake by the interaction of cell surface protein and its aptamer [39]. **E** Application of the DNA nanomesh on the filter membrane [40]. **F** Functionalization of CNT@DNA with DBP molecules containing specific FG and Attachment of multiple NPs onto a CNT [42]. **G** DNA-modified silica and carbon nanotubes were used to amplify the encoding plasmid of interlaced protein in a rolling circle [43]

The introduction of branched DNA is conducive to spanning DNA nanomaterials from multiple dimensions. Branched DNA refers to a specific DNA molecular structure in which a single-stranded DNA molecule has multiple short DNA branches attached to it. These short DNA branches can be homologous or heterologous DNA sequences. Base pair assembly and chemical bonding are the two main methods to construct branched DNA. Base pair assembly includes electrostatic self-assembly and dynamic self-assembly of carefully selected nucleotide sequences. Additionally, the addition of chemical bonds can effectively relieve the tension between branched DNA chains and improve the versatility and heat resistance of products. Yang and his team designed and generated a new supramolecular DNA hydrogel system [24] based on DNA with chemical branches. By introducing chemical branches into DNA molecules, their spatial structure and physical properties can be modified to regulate the properties and behavior of DNA hydrogels. This highly controllable and adjustable method enables the production of hydrogels with diverse properties and functions. The new molecule has better responsiveness and biocompatibility, making it suitable for various biomedical applications. Branched DNA nanomaterials come in three main forms: pure DNA materials, functional DNA fragments, and chemically bonded DNA (Fig. 2A) [25–37].

Consequently, DNA nanomaterials have evolved into a desirable, adaptable, and promising building block for the creation of complex designs. The potential biomedical applications of DNA nanomaterials are also significant. The progress in the field of DNA functional materials is increasingly focused on achieving higher levels of functionality and exploring more intricate avenues. This encompasses the advancement from three-dimensional DNA crystals to crystal devices, the transition from planar structures to multidimensional irregular structures and ordered arrangements, as well as the evolution from microscopic DNA polymers to macroscopic hydrogels.

## Properties of DNA nanomaterials in biomedical applications

DNA nanomaterials have attracted increasing attention in the field of biomedicine due to their biocompatibility, biodegradability, and unique physiological functions. Tian et al. [11] reported a method to create a 3D array-ordered platform using DNA regulation and precise-controlled material voxels (Fig. 2B). This approach enables the precise control and directional arrangement of three-dimensional nanomaterials with higher controllability and repeatability than traditional self-assembly methods. Additionally, it facilitates the combination of different nanomaterials to create new functional materials, expanding the application potential of DNA nanomaterials.

Hu and his team reported SiNP/CNT-DNA nanocomposites with high biocompatibility (Fig. 2C) [38]. Compared to traditional nanomaterials, these composites have several novel features, such as precise drug delivery, enhanced stability and drug loading capacity, and the ability to track and monitor drug distribution and metabolism in vivo using magnetic resonance and fluorescence imaging. These features enhance the safety and efficacy of the drug, making it a promising tool for biomedical research. Wang and his team used molecular docking tools based on a DNA framework to rationally design nanomaterials with stronger stability (Fig. 2D) [39]. This novel DNA nanomaterial has higher stability, reproducibility, and integrity under different conditions, and provides a new tool for DNA enrichment. The combination of this DNA nanomaterial with magnetic nanoparticles allows for specific enrichment of DNA in low-concentration solutions, broadening its potential applications. Wang et al. [40] reported a method of using topoisomerase to keep the stability of DNA nanomaterials (Fig. 2E). This approach transforms the original topological structure of DNA into a DNA nano-reticular structure, increasing its stability and strengthening its enrichment. In biomedical applications, strong enrichment can gather more therapeutic drugs, which have stronger effects [41]. Additional methodologies have been devised for the fabrication of versatile DNA nanomaterials to address specific biomedical requirements, such as precise drug delivery, tumor identification, and drug loading. These strategies include the encapsulation of DNA within carbon nanotubes, incorporating controllable functional groups with arbitrary density (Fig. 2F) [42], as well as functionalizing silica nanoparticles and carbon nanotubes with DNA (Fig. 2G) [43]. These approaches allow for the construction of multifunctional DNA nanomaterials, expanding the scope of their applications in the biomedical field.

In summary, the unique advantages of DNA-guided methods, utilizing the programmable properties of DNA, and the valence control approach, enabling precise control over nanomaterials’ oxidation states, in conjunction with the development of novel and stable DNA nanomaterials and DNA-composite nanomaterials, hold great promise in addressing various biomedical challenges. Specifically, these methods have the potential to facilitate the development of highly sensitive and selective biosensors in biosensing, where DNA-based nanomaterials can serve as imaging agents through direct labeling or as carriers of contrast agents. Furthermore, in the field of diagnostics and therapeutics, they can promote targeted delivery of therapeutic agents.

## Biomedical applications

The successful fabrication of DNA nanomaterials greatly relies on the technical capabilities provided by DNA nanotechnology. Common transformation techniques for DNA nanomaterials include DNA self-assembly, DNA nanotemplate-based methods, DNA nanomanipulation techniques, and chemical modifications of DNA nanomaterials. These techniques enable functional enhancement, diversification, and targeted decoration of DNA nanomaterials. Additionally, research efforts have focused on achieving large-scale production of DNA nanomaterials. With the rapid development of DNA nanotechnology, the growth of DNA nanomaterials has proliferated, which are being utilized in a wide range of biomedical applications. These applications offer exciting possibilities for biosensing, imaging, diagnosis, and therapy of diseases. Specifically, the applications in biosensors are focused on detecting nucleic acids, proteins, and cancer, while the breakthroughs in disease diagnosis and therapy are centered around chemistry, immunology, and gene therapy. In the following sections, examples of biomedical applications of DNA nanomaterials from the last five years in these three broad categories will be presented by me.

### Biosensor

In the biomedical field, biosensors are usually used for detection, and DNA plays a very important role in the construction of biosensors. In recent years, DNA nanomaterials have played an important role in rapid detection strategies, and different nanostructures and design strategies provide abundant molecular libraries for biosensors. DNAzyme-based sensors are synthetic DNA molecules that possess catalytic activity similar to natural enzymes [44]. They offer several advantages over traditional enzyme-based sensors, including higher sensitivity, specificity, and ease of functionalization. Unlike traditional enzyme-based sensors, DNAzyme-based sensors are less affected by environmental factors and do not require complex preparation or costly purification steps. The hyper-branched structure of DNA provides abundant binding sites for target recognition probes and signal molecules, so DNA nanomaterials have high efficiency and sensitive detection ability and are widely used in the field of biosensing [45].

**Fig. 3:**
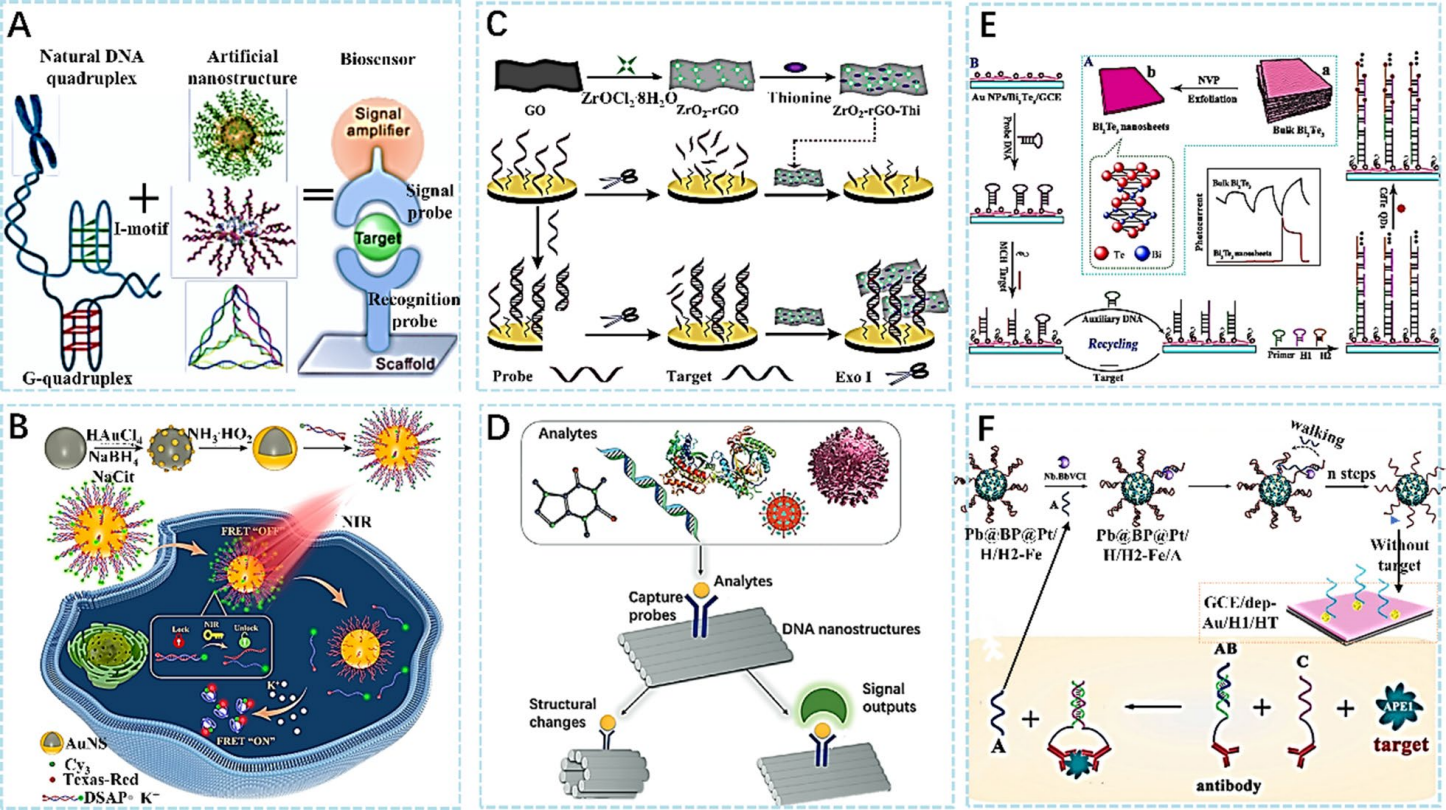
**A** DNA configuration of the biosensor [46]. **B** Intracellular K + sensing based on SNAs fabricated by AuNS with the photothermal property [47]. **C** Schematic illustration of electrochemical nucleic acid fabrication by ZrO_2_-rGO-Thi nanocomposite [48]. **D** Biosensors based on DNA nanotechnology [49]. **E** Synthesis of Bi_2_Te_3_ Nanosheets and Assembly Process of the PEC Biosensor [50]. F construction of this electrochemical biosensor [51]

Generally, the DNA nanostructure used for biosensor construction is called the DNA four-complex project, which is composed of four strands (Fig. 3A) [46]. Here, we can observe a novel class of nanobiomaterials in which a layer of protein is coated onto the surface of nucleic acid nanoparticles. By tuning different nucleic acid sequences and protein types, diverse protein-SNAs with distinct properties can be synthesized, making them suitable for various biological applications. Figure 3B is a DNA nanocomposite that can be used for time-controlled sensing [47]. A nanosystem that can be remotely controlled using a near-infrared (NIR) laser has been designed, which consists of a programmable DNA nanostructure comprising two parts: a ‘lock’ and an ‘unlock’ DNA sequence that can dynamically open and close through nucleic acid hybridization reaction under the influence of NIR laser. Due to its higher sensitivity, this system holds great promise for various biosensing applications. Chen et al. [48] reported an electrochemical DNA biosensor was prepared by using exonuclease and ZrO2-rGO-Thi nanocomposite (Fig. 3C), which provides a simple and effective method for manufacturing the biosensor. The novel electrochemical DNA biosensor is a simple, fast, and reproducible method for detection, which overcomes the limitations of traditional sensors and improves the sensitivity and reliability of detection. In the future, it is expected to be widely used in biomedical analysis and therapeutic diagnosis. Shen et al. [49] made a profound analysis of DNA sensor and their role in the field of therapy. The progress of DNA nanotechnology has paved the way for the development of DNA nanomaterials. Figure 3D is a schematic diagram of a biosensor based on DNA nanotechnology. Yuan and his team used Bi_2_Te_3_ nanosheets to construct a photoelectrochemical biosensor with a DNA amplification strategy, which can be used as a signal amplifier switch [50]. The design of this biosensor utilizes Bi_2_Te_3_ nanosheets as both the light absorber and electron transfer material, quantum dots as the sensitizer, and a DNA amplification strategy to enhance detection sensitivity. By pairing the target molecules with DNA probes, they can form DNA-Bi_2_Te_3_ complexes that aggregate on the surface of Bi_2_Te_3_ and amplify the current signal through the electron transfer process under light illumination. Figure 3E is the schematic diagram of the biosensor developed in this research, which has great application prospects for disease diagnosis and clinical analysis. Then, in 2022, another study also developed a quaternary mesoporous nanosphere using a boron-phosphorus co-doped Pb-Pt alloy (Fig. 3F) [51]. This sensor utilizes the binding of the target molecule APE1 to DNA aptamers, which are immobilized on the surface of quaternary mesoporous spheres, and the interaction between random walk DNA and the electrode surface to achieve signal amplification. The presence of APE1 is detected by measuring the change in the current signal. This method has high sensitivity and selectivity and demonstrates good detection performance in complex biological systems. It provides a new approach to the field of biosensors. Furthermore, it is worth mentioning that, in addition to the biosensing applications mentioned above, DNA nanomaterials also have great potential in drug delivery and therapeutics. DNA nanocarriers, such as DNA origami and DNA hydrogels, have been developed for targeted drug delivery [52], and their high biocompatibility and programmability make them promising candidates for various therapeutic applications.

In summary, DNA nanomaterials have emerged as versatile and powerful tools in the field of biosensors. With their unique properties and diverse designs, DNA nanomaterials offer high sensitivity, specificity, and programmability for biosensing applications. However, there is still much to be explored in terms of integrating DNA nanotechnology into biosensors, and further research is needed to optimize their performance and broaden their application range. Nonetheless, the rapid development of DNA nanomaterials in recent years has opened up exciting opportunities for the development of novel biosensors and has the potential to revolutionize the field of biosensing in the future.

### Therapeutics

DNA nanomaterials have many advantages, making them an important tool in targeted drug delivery, tumor treatment, and disease prevention in biomedicine. DNA materials have been demonstrated to be highly suitable raw materials for the design and construction of biomedical devices. TDNs can achieve anti-inflammatory and antioxidant effects through appropriate design and functionalization. They can easily target specific cells, making them excellent candidates for novel therapeutics [53]. Zhang et al. [54] reported the successful design, preparation, and purification of TDNs, demonstrating their ability to promote cell absorption and maintain biological stability. Recent studies have also explored the potential applications of TDNs in promoting cell proliferation, regulating cell differentiation, facilitating cell migration, and supporting the bioactivity of the extracellular matrix (ECM). TDNs also have broad potential in wound repair and regeneration [55].

**Fig. 4:**
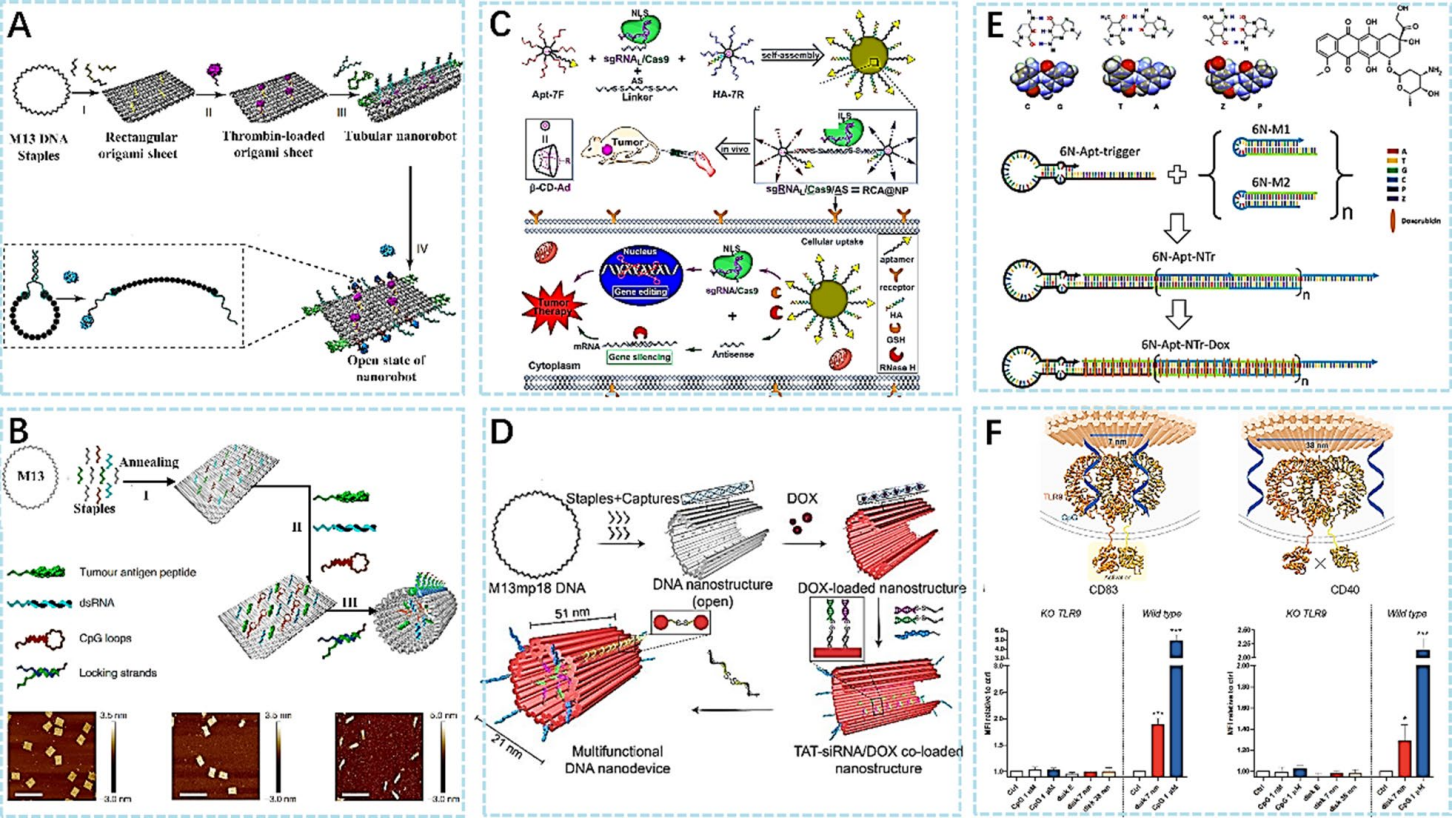
**A** Design and characterization of thrombin-functionalized DNA nanorobot [56]. **B** Design and characterization of the antigen/adjuvant-functionalized DNA nanodevice vaccine [57]. **C** Schematic representation of the sgRNA/Cas9/antisense complex in DNA nanoplatforms for tumor therapy [35]. **D** Design of a multifunctional tubular DNA nanodevice [58]. **E** Key components of AEGIS aptamer-nanotrain assembly [59]. **F** Two CpG molecules have corresponding activation effects at 7 nm and 38 nm apart and the cubic average plot of experimental results [60]

DNA nanorobot is a special type of DNA nanomaterial that has emerged in recent years. Li et al. [56] have successfully constructed DNA robots that can be programmed to target tumor cells and release drugs in response to specific molecular triggers in the tumor tissue, inhibiting tumor growth. Figure 4A shows a schematic diagram of a DNA nanorobot carrying a thrombin-carrying agent. Recently, a DNA nanodevice vaccine was also reported [57]. The vaccine triggers T cell activation and cancer cell toxicity through molecular adjuvant and antigen peptides assembled in the lumen of DNA origami (Fig. 4B), producing a long-term T cell response to avoid tumor recurrence. Effective assembly of DNA nanostructures is crucial in biomolecular therapy. Liu et al. [35] introduced a strategy of using branched DNA to construct nanoplatforms (Fig. 4C). This DNA-based self-assembling platform is constructed from two types of branched DNA: core branch DNA and peripheral branch DNA. The core branch DNA contains two complementary sequences, one for binding to the target gene and the other for the carrier molecule, as well as additional short sequences for connecting to the peripheral branch DNA. The peripheral branch DNA contains complementary sequences that match the connecting sequence of the core branch DNA, as well as additional short sequences for binding nucleic acid drugs such as sgRNA, Cas9, and ASO. Some studies have also constructed DNA nanodevices based on small interfering RNA, which can insert doxorubicin (DOX) into DNA double strands [54]. DNA molecules are used to construct nanodevices with a hollow tubular structure that can protect chemotherapy drugs and guide siRNA to tumor cells. By simultaneously targeting multiple therapeutic targets in cells, this nanodevice can improve treatment efficacy, reduce drug dosage, and minimize side effects. However, this DNA-based nanomaterial device is still in the experimental stage. Wang et al. [58] achieved a mechanism by constructing DNA origami structures loaded with siRNA and coassembling them with multiple functional moieties, including tumor-penetrating ligands and stimuli-responsive DNA locks. This mechanism allows for the protection of siRNA upon localization to tumor cells and its triggered release at an appropriate time. Figure 4D illustrates the schematic diagram of a DNA nanodevice. In addition, the research team utilized the functional roles of P:Z pairs to successfully construct a structure called the 'nanotrains', which were designed for efficient delivery of the anticancer drug Doxorubicin (Dox). Figure 4E illustrates the formation process of this structure and the corresponding workflow [59]. The nanomaterial is constructed from six-base DNA molecules and has an internal cavity that can accommodate multiple DOX molecules. Through specific binding with ASGPR, the nanodevice accurately recognizes liver cancer cells and releases DOX to achieve targeted treatment of liver cancer cells, reducing damage to normal cells, and enhancing the local concentration of the drug, thus improving therapeutic efficacy. Additionally, DNA nanomaterials can also fine-tune the immune system by spatially controlling the activation of Toll-like receptor 9 (TLR9), thereby inducing an immune response in the body [60] (Fig. 4F). Both the DNA nanomaterial and CpG sequences were subjected to modulation. Various connecting sequences were synthesized to attach CpG sequences to the DNA nanomaterial. It is noteworthy that the connecting regions of these sequences were not phosphorothioated, and their activity and impact have not been tested. In addition to controlling the spatial relationship between CpG sequences, the connecting sequences represent another variable that needs to be considered. Therefore, when interpreting the research results, it is important to take into account both spatial control and the presence and influence of the connecting sequences.

While numerous studies have demonstrated the considerable potential of DNA nanomaterials in the field of biomedical diagnosis and treatment, it is important to acknowledge that several obstacles and hurdles remain. Firstly, biomedical applications encompass a broad range of areas, and the internal biological environment is characterized by its intricacy and limited scale. Secondly, prior to experimental trials, DNA nanomaterials require scaling up from the micro to macro levels in terms of manufacturing scale, which is a protracted and intricate process. Therefore, in the future, we need to continually experiment with the interaction between different DNA nanomaterials and organisms and design optimal strategies to reduce the time and cost required for experimentation.

### Bioimaging

**Fig. 5:**
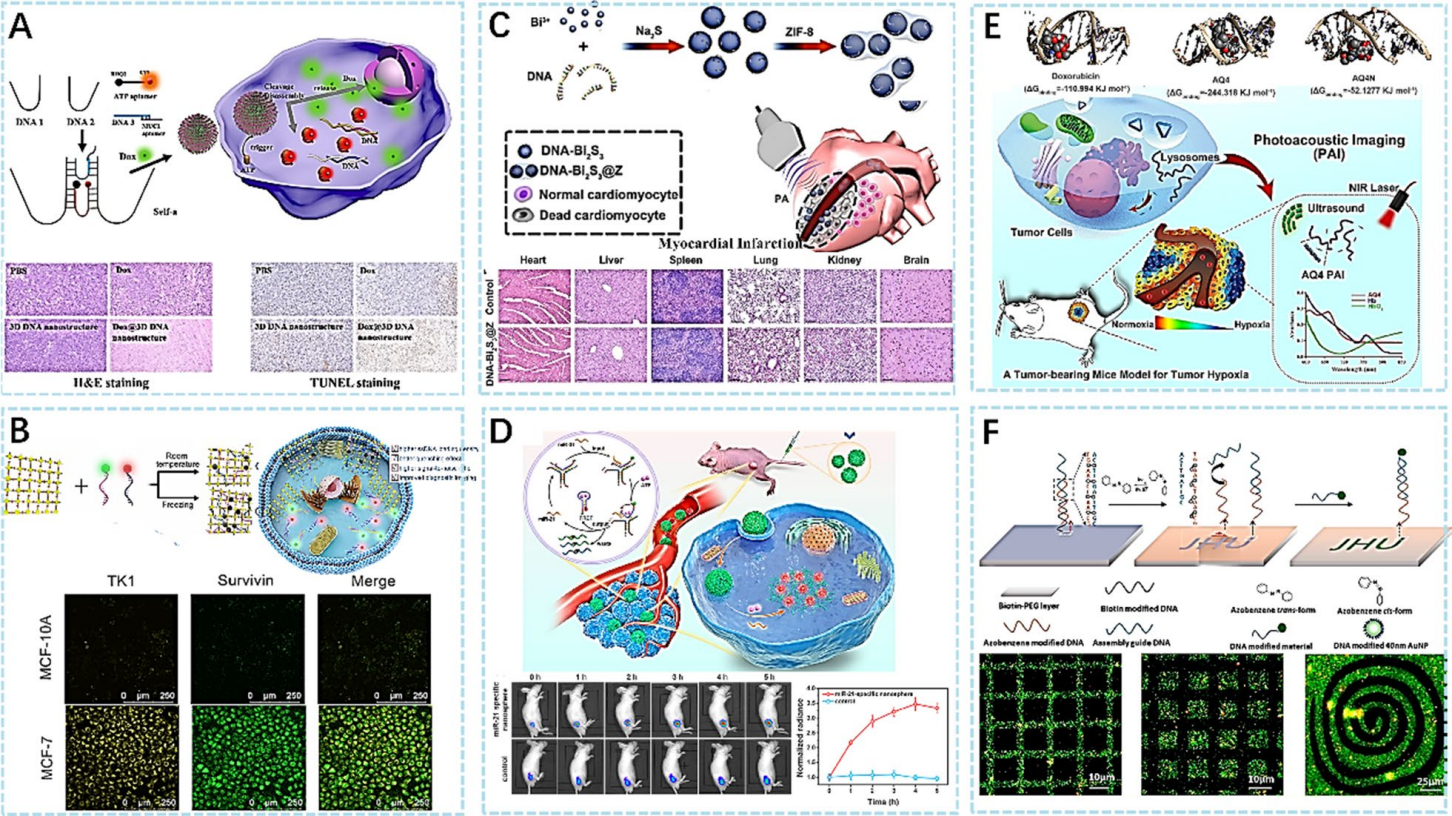
**A** ATP triggered Drug Release for Tumor Fluorescence Imaging Analysis and Targeted Drug Delivery [61]. **B** Cancer diagnostic imaging using COF NPs and COF-DNA bicolor nanoprobes is shown schematically in the illustration, along with confocal images of MCF-10A and MCF-7 cells [62]. **C** An example of a schematic showing how to make DNA-Bi2S3@Z NPs for PA imaging of MI with increased contrast and H&E-stained images of a large organ [63]. **D** ATP-fueled cyclic dissociation and fluorescent pictures of MCF-7 tumor-bearing mice were used to demonstrate the DNA nanosphere’s mode of operation for intracellular amplified miR-21 imaging [65]. **E** Doxorubicin/AQ4/AQ4N interactions with DNA double helix are shown schematically in this picture using a chemotherapeutic drug [62]. **F** A square array coupled with AuNPs and a light-driven DNA-POINT network for self-assembly and symmetric patterns [67]

In recent years, DNA nanomaterials have been applied in various fields, including biomedical imaging. Accurate identification of diseased tissue is crucial for effective treatment, and DNA nanostructures are promising imaging materials due to their structural designability and sequence diversity. For example, Jiang et al. [61] designed a self-assembled 3D DNA nanostructure drug delivery system for fluorescence imaging analysis of tumors and targeted drug delivery to tumor cells (Fig. 5A). This 3D nanoscale structure consists of two functional regions, one for fluorescence imaging and the other for drug delivery, and can release drugs through ATP stimulation, achieving precise treatment and fluorescence imaging of tumors. In addition, COF-DNA bicolor probes have been developed [62] to provide new detection perspectives for a variety of biological assays (Fig. 5B). This nanoprobe is composed of two parts: one is a DNA oligonucleotide sequence that can specifically hybridize with tumor-related mRNA, and the other is a COF material for fluorescence imaging. It can efficiently detect and image tumor tissues and tumor-related mRNA in mice while exhibiting good biocompatibility and low cytotoxicity.

It is worth noting that nanoprobes prepared by cryoprobes have better performance. DNA-templated ultramicroscopic bismuth sulfide (Bi_2_S_3_) nanoparticles (NPs) have been developed as photoacoustic probes for imaging by Zhao et al. [63] and his team (Fig. 5C). They were successfully used in the imaging of myocardial infarction, exhibiting excellent biocompatibility, stability, and high photoacoustic imaging performance. Similarly, DNA-templated copper nanoparticles were used to stain the nuclei of human colon cell lines [64], providing a theoretical reference for other DNA-templated nanomaterials and improving the bioimaging process. Additionally, self-assembled DNA nanospheres have been utilized to amplify miRNA in mice imaging by Wang et al. [65] (Fig. 5D). This experiment demonstrated that programmable DNA nanospheres have the capability to detect the expression of murine miRNA cancer cells with size control, self-assembly capabilities, excellent ATP-driven cyclic dissociation, and reassembly properties for imaging applications. It has the advantage of efficient signal amplification and imaging of microRNAs, making it a promising tool for potential biomedical applications. Overall, DNA nanomaterials have great potential for biomedical imaging applications, but more research is needed to improve their performance and optimize their design for different imaging modalities.

Different from previous studies, Zeng et al. [66] utilized DNA origami to create a photoacoustic contrast agent and investigated the mechanism of chemotherapy on tumor hypoxia (Fig. 5E). In addition to their applications in imaging and cancer therapy, DNA nanomaterials also hold promise in topographic optical imprinting. Liang et al. [67] utilized DNA patterns, called DNA points, for the rapid optical patterning of large, geometrically complex surfaces using light-responsive DNA (Fig. 5F). This study also demonstrated the scanning mode of DNA points, paving the way for the fabrication of biochips. Qin et al. [68] used DNA-modified gold nanoparticles for the identification of Mycoplasma pneumonia, a simpler and more rapid detection method. These nanoparticles can be used for the sensitive and quantitative detection of Mycoplasma pneumoniae and have the potential for clinical imaging applications. The aforementioned studies present the latest advancements in the use of DNA nanomaterials in biomedicine in 2022. While DNA nanomaterials are currently widely used in imaging and tumor therapy, they hold potential for flexible applications in various fields in the future.

## Conclusion

DNA molecules can combine with unique materials to create a diverse range of nanostructures, offering broad applications across various aspects of biomedicine. The above examples demonstrate the vast potential of DNA nanomaterials in biomedicine, including successful tumor treatment in animal models and promising therapeutic potential. However, it is crucial to acknowledge that the limited stability of these materials under physiological conditions, along with their potential immunogenicity, presents inherent risks in the context of utilizing DNA nanostructures in vivo. These risks encompass various aspects, including impaired biological function, inadequate biodistribution, and a shortened circulation time. Therefore, when evaluating the stability, biodistribution, and pharmacokinetics of DNA nanostructures in vivo, these potential challenges must be taken into careful consideration to ensure their effective and safe application in biomedical settings. Over the last three years, there have been rapid developments in the field of biosensors, disease diagnosis, and treatment, with much of the research focused on gene and drug delivery, bioimaging, biosensors, and diagnostics. DNA nanostructures have been extensively studied in the biomedical field, with continuous advances in design, shape, and size. However, clinical research on DNA nanocarriers has not yet been conducted, and further research is necessary to explore their potential applications.

This chapter discusses the current status and future development trends of DNA nanomaterials in biomedical applications, including:

A) More research is needed to determine crucial details about possible biomedical applications of DNA nanoagents. The in vivo parameters of DNA nanocarriers that need to be examined include their circulation half-life, pharmacokinetics, size- and shape-dependent properties for passive tumor targeting, uptake, intracellular fate, and clearance process. Additionally, active ligand-based targeting strategies should be investigated to enhance the specificity and efficacy of DNA nanoagents in biomedical applications.
B) PEGylation or other polymer coatings can increase stability and systemic circulation by functionalizing structures with positively charged molecules. This approach helps to enhance the overall performance and behavior of DNA nanostructures in biological systems.
C) Tailored DNA nanocarriers based on smart DNA architecture can be anticipated to provide secure and effective individualized applications for cancer treatment in the future given the growing knowledge of DNA structure design, DNA material behaviors in vivo, and molecular interactions with cancer.
D) DNA nanostructures exhibit remarkable controllability, high drug-loading capacity, excellent biocompatibility, as well as precise targeting and efficient tissue penetration in biomedical applications.
E) The limited yield of DNA templates may result in increased costs. In the fabrication process of DNA nanomaterials, DNA templates are typically necessary for controlling the desired structures and sequences. Better methods are needed to obtain DNA templates, and the current synthetic processes must be simplified to increase product yields.
F) Despite significant efforts in the medicinal uses of DNA materials, long-term biological stability remains a concern. The precise mechanism of DNA nanostructures in vivo is not yet fully understood, highlighting the need for more in-depth investigations of DNA stability both in vitro and in vivo, calling for interdisciplinary study.

In summary, significant progress has been made in the field of DNA nanotechnology in recent years, and DNA nanomaterials have become promising tools for various biomedical applications. These programmable and multifunctional nanomaterials possess unique characteristics such as high biocompatibility, self-assembly, programmability, and precise molecular recognition, making them highly attractive in various biomedical applications. With further research and development, DNA nanomaterials have the potential to revolutionize the field of biomedical research and improve human health. In the future, DNA nanomaterials can be used to further explore the mechanisms of human disease and to dissect cellular metabolism levels. Additionally, they could be introduced to the fields of precision therapy, gene therapy, biomimetic materials, and artificial intelligence.

## Data Availability

Not applicable.

## References

[1] Malik R, Johnson RE, Prakash L, Prakash S, Ubarretxena-Belandia I, Aggarwal AK (2022). Cryo-EM structure of translesion DNA synthesis polymerase ζ with a base pair mismatch. Nat Commun.

[2] Tan S, McCoy A (2020). James Dewey Watson (1928–): co-discoverer of the structure of DNA. Singapore Med J.

[3] Ngai CK, Lam SL, Lee HK, Guo P (2022). A purine and a backbone discontinuous site alter the structure and thermal stability of DNA minidumbbells containing two pentaloops. FEBS Lett.

[4] Dai P, Williams CT, Witucki AM, Rudge DW (2021). Rosalind Franklin and the discovery of the structure of DNA: using historical narratives to help students understand nature of science. Sci Educ.

[5] Dong Y, Yao C, Zhu Y, Yang L, Luo D, Yang D (2020). DNA functional materials assembled from branched DNA: design, synthesis, and applications. Chem Rev.

[6] Seeman NC (1998). DNA nanotechnology: novel DNA constructions. Annu Rev Biophys Biomol Struct.

[7] Wang P, Xiao M, Pei H (2021). Biomineralized DNA nanospheres by metal organic framework for enhanced chemodynamic therapy. Chem Eng J.

[8] Qi H, Xu Y, Hu P, Yao C, Yang D (2022). Construction and applications of DNA nanomaterials in cancer therapy. Chin Chem Lett.

[9] Llewellyn SV, Niemeijer M, Nymark P (2021). In vitro three-dimensional liver models for nanomaterial DNA damage assessment. Small.

[10] Baker YR, Yuan L, Chen J (2021). Expanding the chemical functionality of DNA nanomaterials generated by rolling circle amplification. Nucleic Acids Res.

[11] Tian Y, Lhermitte JR, Bai L (2020). Ordered three-dimensional nanomaterials using DNA-prescribed and valence-controlled material voxels. Nat Mater.

[12] Suo Z, Chen J, Hu Z, Liu Y, Xing F, Feng L (2018). Recent advances in novel DNA guiding nanofabrication and nanotechnology. Nanofabrication.

[13] Baig MMFA, Dissanayaka WL, Zhang C (2021). 2D DNA nanoporous scaffold promotes osteogenic differentiation of pre-osteoblasts. Int J Biol Macromol.

[14] Lu H, Hailin T, Yi X, Wang J (2020). Three-dimensional DNA nanomachine combined with toehold-mediated strand displacement reaction for sensitive electrochemical detection of miRNA. Langmuir.

[15] Ma W, Zhan Y, Zhang Y, Mao C, Xie X, Lin Y (2021). The biological applications of DNA nanomaterials: current challenges and future directions. Signal Transduct Target Ther.

[16] Toivari M, Nygård Y, Kumpula EP (2012). Metabolic engineering of *Saccharomyces cerevisiae* for bioconversion of d-xylose to d-xylonate. Metab Eng.

[17] Fu J, Liu M, Liu Y, Yan H (2012). Spatially-interactive biomolecular networks organized by nucleic acid nanostructures. Acc Chem Res.

[18] Wang X, Chandrasekaran AR, Shen Z (2019). Paranemic crossover DNA: there and back again. Chem Rev.

[19] Dhar S, Gu FX, Langer R, Farokhzad OC, Lippard SJ (2008). Targeted delivery of cisplatin to prostate cancer cells by aptamer functionalized Pt(IV) prodrug-PLGA–PEG nanoparticles. Proc Natl Acad Sci USA.

[20] Lee H, Lytton-Jean AKR, Chen Y (2012). Molecularly self-assembled nucleic acid nanoparticles for targeted in vivo siRNA delivery. Nat Nanotechnol.

[21] Jiang S, Eltoukhy AA, Love KT, Langer R, Anderson DG (2013). Lipidoid-coated iron oxide nanoparticles for efficient DNA and siRNA delivery. Nano Lett.

[22] Wei T, Cheng Q, Farbiak L, Anderson DG, Langer R, Siegwart DJ (2020). Delivery of tissue-targeted scalpels: opportunities and challenges for in vivo CRISPR/Cas-based genome editing. ACS Nano.

[23] Liu F, Shang Y, Wang Z, Jiao Y, Li N, Ding B (2020). DNA origami directed fabrication of shape-controllable nanomaterials. APL Mater.

[24] Yang B, Zhao Z, Pan Y (2021). Shear-thinning and designable responsive supramolecular DNA hydrogels based on chemically branched DNA. ACS Appl Mater Interfaces.

[25] He Y, Chen Y, Liu H, Ribbe AE, Mao C (2005). Self-assembly of hexagonal DNA two-dimensional (2D) arrays. J Am Chem Soc.

[26] Cheng E, Xing Y, Chen P (2009). A pH-triggered, fast-responding DNA hydrogel. Angew Chem.

[27] Zheng J, Birktoft JJ, Chen Y (2009). From molecular to macroscopic via the rational design of a self-assembled 3D DNA crystal. Nature.

[28] Iinuma R, Ke Y, Jungmann R, Schlichthaerle T, Woehrstein JB, Yin P (2014). Polyhedra self-ASSEMBLED from DNA tripods and characterized with 3D DNA-PAINT. Science.

[29] Zhang F, Jiang S, Wu S (2015). Complex wireframe DNA origami nanostructures with multi-arm junction vertices. Nat Nanotechnol.

[30] Hong F, Jiang S, Wang T, Liu Y, Yan H (2016). 3D framework DNA origami with layered crossovers. Angew Chem Int Ed.

[31] Kwon PS, Ren S, Kwon SJ (2020). Designer DNA architecture offers precise and multivalent spatial pattern-recognition for viral sensing and inhibition. Nat Chem.

[32] Wang W, Chen S, An B (2019). Complex wireframe DNA nanostructures from simple building blocks. Nat Commun.

[33] Yang H, McLaughlin CK, Aldaye FA (2009). Metal–nucleic acid cages. Nat Chem.

[34] Finke A, Bußkamp H, Manea M, Marx A (2016). Designer extracellular matrix based on DNA-peptide networks generated by polymerase chain reaction. Angew Chem Int Ed.

[35] Liu J, Wu T, Lu X (2019). A self-assembled platform based on branched DNA for sgRNA/Cas9/antisense delivery. J Am Chem Soc.

[36] Tapio K, Bald I (2020). The potential of DNA origami to build multifunctional materials. Multifunct Mater.

[37] Ma J, Xu J (2021). Logic gates in nanoscale based on interaction of thiolated DNA with AuNPs and strand displacement. Biosystems.

[38] Hu Y, Niemeyer CM (2020). Designer DNA–silica/carbon nanotube nanocomposites for traceable and targeted drug delivery. J Mater Chem B.

[39] Wang C, Yu Y, Irfan M (2020). Rational design of DNA framework-based hybrid nanomaterials for anticancer drug delivery. Small.

[40] Wang X, Yu J, Lan W (2020). Novel stable DNA nanoscale material and its application on specific enrichment of DNA. ACS Appl Mater Interfaces.

[41] Tian T, Li Y, Lin Y (2022). Prospects and challenges of dynamic DNA nanostructures in biomedical applications. Bone Res.

[42] Kim K, Yoon S, Chang J (2020). Multifunctional heterogeneous carbon nanotube nanocomposites assembled by DNA-binding peptide anchors. Small.

[43] Schipperges A, Hu Y, Moench S (2021). Formulation of DNA nanocomposites: towards functional materials for protein expression. Polymers.

[44] Yu R, Wang R, Wang Z, Zhu Q, Dai Z (2021). Applications of DNA-nanozyme-based sensors. Analyst.

[45] Bhanjadeo MM, Nayak AK, Subudhi U (2017). Surface-assisted DNA self-assembly: an enzyme-free strategy towards formation of branched DNA lattice. Biochem Biophys Res Commun.

[46] Samanta D, Ebrahimi SB, Kusmierz CD, Cheng HF, Mirkin CA (2020). Protein spherical nucleic acids for live-cell chemical analysis. J Am Chem Soc.

[47] Cui MR, Chen LX, Li XL, Xu JJ, Chen HY (2020). NIR remote-controlled “lock–unlock” nanosystem for imaging potassium ions in living cells. Anal Chem.

[48] Chen Z, Liu X, Liu D, Li F, Wang L, Liu S (2020). Ultrasensitive electrochemical DNA biosensor fabrication by coupling an integral multifunctional zirconia-reduced graphene oxide-thionine nanocomposite and exonuclease I-assisted cleavage. Front Chem.

[49] Shen L, Wang P, Ke Y (2021). DNA nanotechnology-based biosensors and therapeutics. Adv Healthc Mater.

[50] Yuan Y, Hu T, Zhong X, Zhu M, Chai Y, Yuan R (2020). Highly sensitive photoelectrochemical biosensor based on quantum dots sensitizing Bi 2 Te 3 nanosheets and DNA-amplifying strategies. ACS Appl Mater Interfaces.

[51] Wei Q, Teng Z, Luo X (2022). Incorporating quaternary mesoporous nanospheres and DNA stochastic nanowalkers into a signal amplified switch: a highly sensitive electrochemical assay for APE1. Sens Actuators B Chem.

[52] Sabari JK, Offin M, Stephens D (2019). A prospective study of circulating tumor DNA to guide matched targeted therapy in lung cancers. JNCI J Natl Cancer Inst.

[53] Zhang Q, Lin S, Shi S (2018). Anti-inflammatory and antioxidative effects of tetrahedral DNA nanostructures via the modulation of macrophage responses. ACS Appl Mater Interfaces.

[54] Zhang T, Tian T, Zhou R (2020). Design, fabrication and applications of tetrahedral DNA nanostructure-based multifunctional complexes in drug delivery and biomedical treatment. Nat Protoc.

[55] Dou Y, Cui W, Yang X, Lin Y, Ma X, Cai X (2022). Applications of tetrahedral DNA nanostructures in wound repair and tissue regeneration. Burns Trauma.

[56] Li S, Jiang Q, Liu S (2018). A DNA nanorobot functions as a cancer therapeutic in response to a molecular trigger in vivo. Nat Biotechnol.

[57] Liu S, Jiang Q, Zhao X (2021). A DNA nanodevice-based vaccine for cancer immunotherapy. Nat Mater.

[58] Wang Z, Song L, Liu Q (2021). A tubular DNA nanodevice as a siRNA/chemo-drug co-delivery vehicle for combined cancer therapy. Angew Chem Int Ed.

[59] Zhang L, Wang S, Yang Z (2020). An aptamer-nanotrain assembled from six-letter DNA delivers doxorubicin selectively to liver cancer cells. Angew Chem Int Ed.

[60] Comberlato A, Koga MM, Nüssing S, Parish IA, Bastings MMC (2022). Spatially controlled activation of toll-like receptor 9 with DNA nanomaterials. Nano Lett.

[61] Jiang Y, Zhou H, Zhao W, Zhang S (2022). ATP-triggered drug release of self-assembled 3D DNA nanostructures for fluorescence imaging and tumor therapy. Anal Chem.

[62] Gao P, Yin J, Wang M (2022). COF-DNA bicolor nanoprobes for imaging tumor-associated mRNAs in living cells. Anal Chem.

[63] Zhao P, Li B, Li Y, Chen L, Wang H, Ye L (2022). DNA-Templated ultrasmall bismuth sulfide nanoparticles for photoacoustic imaging of myocardial infarction. J Colloid Interface Sci.

[64] Kim S, Kim JH, Kwon WY (2019). Synthesis of DNA-templated copper nanoparticles with enhanced fluorescence stability for cellular imaging. Microchim Acta.

[65] Wang J, Li J, Chen Y (2022). Size-controllable and self-assembled DNA nanosphere for amplified microRNA imaging through ATP-fueled cyclic dissociation. Nano Lett.

[66] Zeng Y, Chang P, Ma J (2022). DNA origami-anthraquinone hybrid nanostructures for in vivo quantitative monitoring of the progression of tumor hypoxia affected by chemotherapy. ACS Appl Mater Interfaces.

[67] Liang L, Jia S, Barman I (2022). DNA-POINT: DNA patterning of optical imprint for nanomaterials topography. ACS Appl Mater Interfaces.

[68] Qin D, Gong Q, Li X (2022). Identification of Mycoplasma pneumoniae by DNA-modified gold nanomaterials in a colorimetric assay. Biotechnol Appl Biochem.

